# Bayesian Spatial Semi-Parametric Modeling of HIV Variation in Kenya

**DOI:** 10.1371/journal.pone.0103299

**Published:** 2014-07-25

**Authors:** Oscar Ngesa, Henry Mwambi, Thomas Achia

**Affiliations:** 1 School of Mathematics, Statistics and Computer Science, University of KwaZulu-Natal, Pietermaritzburg, KwaZulu-Natal, South Africa; 2 Division of Epidemiology and Biostatistics, University of Witwatersrand, Johannesburg, Gauteng, South Africa; University of Athens, Medical School, Greece

## Abstract

Spatial statistics has seen rapid application in many fields, especially epidemiology and public health. Many studies, nonetheless, make limited use of the geographical location information and also usually assume that the covariates, which are related to the response variable, have linear effects. We develop a Bayesian semi-parametric regression model for HIV prevalence data. Model estimation and inference is based on fully Bayesian approach via Markov Chain Monte Carlo (McMC). The model is applied to HIV prevalence data among men in Kenya, derived from the Kenya AIDS indicator survey, with n = 3,662. Past studies have concluded that HIV infection has a nonlinear association with age. In this study a smooth function based on penalized regression splines is used to estimate this nonlinear effect. Other covariates were assumed to have a linear effect. Spatial references to the counties were modeled as both structured and unstructured spatial effects. We observe that circumcision reduces the risk of HIV infection. The results also indicate that men in the urban areas were more likely to be infected by HIV as compared to their rural counterpart. Men with higher education had the lowest risk of HIV infection. A nonlinear relationship between HIV infection and age was established. Risk of HIV infection increases with age up to the age of 40 then declines with increase in age. Men who had STI in the last 12 months were more likely to be infected with HIV. Also men who had ever used a condom were found to have higher likelihood to be infected by HIV. A significant spatial variation of HIV infection in Kenya was also established. The study shows the practicality and flexibility of Bayesian semi-parametric regression model in analyzing epidemiological data.

## Introduction

Globally, people living with HIV were estimated to be 35.3 million in 2012 with 2.2 million new infections. It is estimated that over two thirds of all persons living with HIV are in sub-Saharan Africa [1]. Furthermore, this region also has the highest prevalence and HIV infection incidence in the entire world [1].

Many prevention strategies are being employed to minimize new infections and to improve the living standards of HIV-infected persons. Notably, male circumcision campaigns [2]–[4], introduction of tropical microbicides [5]–[6], vaccine initiatives [7]–[9] and the roll out of antiretroviral therapy [10].

Monitoring the prevalence of HIV in a country using national averages is important, especially for assessing the HIV trend at national level over time and for comparison purposes between countries. However, the approaches can facade HIV prevalence variability among administration units in the country [11]. Subsequently, policies and interventions developed from these approaches might have no effect at the grassroots level.

In Kenya, HIV prevalence in 2007 was estimated to be approximately at 7.1% among adults aged 15–64 years [13]. HIV prevalence varies considerably between sub-regions in the country. Counties like Bungoma, Embu, Isiolo, Kajiado, Machakos, Meru, Wajir and West Pokot having a prevalence of less than 3% while counties close to the Lake Victoria region, in the West having prevalence ranging between 13% and 25% [13]. The dissimilarities in these regions may be explained by cultural factors, demographic factors and socioeconomic factors [14]–[15].

A proper understanding of the spatial component of HIV among the administrative units in a country is key to structuring, developing and implementing apposite strategies that will have an effect on people at the local administrative units [16].

HIV prevalence in Kenya also varies significantly by sex and age. HIV prevalence among women in Kenya is 6.9% and 4.4% among men [12]. HIV prevalence by age has a nonlinear relationship, assuming an inverted U-shape [14], [17]. In particular, HIV prevalence is low among people below the age of 18, the prevalence increases up to the age of 35–40 then starts declining with increase in age.

Several studies on spatial analysis of HIV prevalence at lower administration units in Kenya have been based on proportions [17], [18]. This prevalence estimates and corresponding maps are prone to instability, hence unreliable due to small sample sizes from these administrative units. Some studies have applied spatial smoothing techniques to circumnavigate this sparse data problem [19]. However, these studies have inherently assumed that all covariates in the study have a linear relationship with the response variable. This linear relationship might not be valid for some variables. As noted earlier, HIV prevalence has a nonlinear relationship with age of an individual.

A primary objective of this study is to develop and apply flexible models to capture this nonlinear nature of some covariates while still accounting for the spatial heterogeneity. The study proposes a spatial semi-parametric model based on penalized regression spline to model HIV prevalence data among men, extracted from the Kenya AIDS Indicator Survey (2007).

## Methods

### Data

The Kenya AIDS Indicator Survey (KAIS) was carried out by the Government of Kenya with financial support from United States President's Emergency Plan for AIDS Relief (PEPFAR) and United Nations (UN). The key objective of survey was to collect high quality data on the prevalence of HIV and Sexually Transmitted Infections (STI) among adults, and to assess knowledge of HIV and STI in the populations.

The survey collected a representative sample of households selected from the eight provinces in the country. It involved men and women in the age of 15–64 years. Two questionnaires were used in the survey. A household questionnaire which was used to collected information about the household head and the characteristics of the dwelling place. The second one, the individual questionnaire collected information from men and women aged 15–64 years, about their demographic characteristics, and their knowledge on HIV and STI. Each individual was then asked for consent to provide a venous blood sample for HIV and HSV-2 testing. Readers are referred to the final survey report regarding survey methodologies used in collecting the data [13]. Even though a new round of KAIS, 2012 [12] has been done, this study uses the 2007 data since the final release of this new study has not been made hence the data was not availlable for use. In this study we use the men's data from this survey. In total, 3,662 men who provided venous blood for testing and also had full covariate information were used in the analysis. In the data, age was captured as both categorical and continous while all the other covariates were continuous. Table 1 gives an initial exploratory analysis used to identify variables that are significantly associated with HIV infection. The variables were categorised in to four groups, namely, demographic, social, biological and behavioural.

**Table 1:** Exploratory data analysis.

Variable	p-value	Unadjusted OR	95% CI for OR
**Demographic characteristics**			
*Age(ref 15–19)*	0.000	1	
*20–24*	0.009	0.337	(0.149,0.759)
*25–29*	0.363	0.714	(0.346,1.474)
*30–34*	0.017	2.217	(1.151,4.269)
*35–39*	0.004	2.62	(1.362,5.04)
*40–44*	0.001	3.024	(1.571,5.823)
*45–49*	0.001	3.231	(1.668,6.257)
*50–54*	0.013	2.362	(1.195,4.67)
*55–59*	0.022	2.284	(1.127,4.628)
*60–64*	0.796	0.894	(0.383,2.09)
Place of residence *(Ref Rural)*		1	
*Urban*	0.016	1.326	(1.055,1.668)
**Social characteristics**			
Number of children dead (*ref Never given birth*)	0.658	1	
*no child died*	0.999	0.491	(0.310,0.803)
*one child died*	0.802	0.763	(0.093,6.294)
*more than one child died*	0.970	0.960	(0.116,7.928)
Education level (*ref None*)	0.047	1	
*Primary*	0.177	1.183	(0.927,1.511)
*Secondary*	0.050	1.108	(0.047,0.079)
*Higher*	0.047	0.721	(0.476,0.991)
Wealth quantile (*ref Poorest*)	0.889	1	
*Second*	0.750	1.060	(0.741,1.517)
*Middle*	0.938	1.014	(0.710,1.449)
*Fourth*	0.954	0.990	(0.693,1.414)
*Richest*	0.430	1.141	(0.822,1.584)
Marital status (*ref Married,1partner*)	0.000	1	
*Married, +2partner*	0.002	1.762	(1.232,2.520)
*Divorced/Separated*	0.831	1.053	(0.656,1.689)
*Widowed*	0.000	3.318	(1.925,5.720)
*Never Married*	0.000	0.310	(0.230,0.420)
Age at first sex (*ref Never had sex*)	0.000	1	
*under 11*	0.302	1.768	(0.599,5.22)
*between 12–14*	0.000	6.678	(3.393,13.144)
*between 15–17*	0.000	6.264	(3.282,11.953)
*over 18*	0.000	5.400	(2.836,10.284)
Perceived risk of HIV (*ref No risk*)	0.010	1	
*Small Risk*	0.001	0.424	(0.253,0.711)
*Moderate Risk*	0.005	0.501	(0.308,0.813)
*Great Risk*	0.891	1.038	(0.612,1.759)
**Biological characteristics**			
Circumcision status (*ref circumcised*)		1	
*Not circumcised*	0.000	4.344	(3.479,5.423)
Had STI in the last 12 months (*ref Yes*)		1	
*No*	0.003	0.464	(0.28,0.769)
**Behavioural characteristics**			
Ever used Condom (*ref Yes*)		1	
*No*	0.000	0.641	(0.505,0.814)
Paid for sex (*ref Yes*)		1	
*No*	0.430	0.712	(0.306,1.657)
Freq of travel away (*ref Did not stay away*)	0.089	1	
*stayed away 1–2 times*	0.127	1.233	(0.942,1.613)
*stayed away 3–5 times*	0.024	1.415	(1.047,1.914)
*stayed away 6–10 times*	0.255	1.290	(0.832,1.999)
*stayed away >11 times*	0.408	0.797	(0.466,1.363)

From this initial analysis, the following variables were found to be associated with HIV infection and were included in subsequent analyses: place of residence, age, education level, marital status, age at first sex, perceived risk of HIV, circumcision status, if had STI in the last 12 months and if ever used a condom. Further from this initial analysis, it was evident that age had a nonlinear effect on HIV infection, hence its continous form (mean  = 33.70, SD = 13. 45) was used in the subsequent analyses.

### Ethics Statement

Ethical clearance was granted by the institutional review board of the Kenya Medical Research Institute (KEMRI) and the US Centers for Disease Control and Prevention. The consent procedure, highlighted below, was approved by these two bodies.

Participants provided separate informed oral consent for interviews, blood draws and blood storage and, the interviewer signed the consent form to indicate whether or not consent was given for each part. An oral informed consent was given for participants in the age of 18–64 while for minors, in the age group 15–17, oral informed consent was obtained from a parent/guardian or other adult responsible for the youth's health and welfare before the youth was asked for his/her consent. Only after the parent or guardian had agreed, was when the consent was asked of the adolescent.

Investigators in the study got a waiver of documentation of informed consent for all participants due to the fact that the research presented very minimal risk of harm to the individuals. The waiver did not adversely affect the rights and welfare of the participants, and the survey involved no procedures for which written consent is normally required outside the research context in Kenya.

### Statistical model

Let 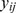 be the HIV status of individual 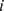 in county 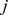. 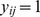 if individual 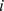 in county 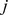 is HIV positive and zero otherwise.

The vector 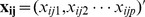 contains 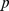 continuous independent random variables and 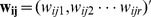 contains 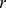
categorical independent random variables with first component accounting for the intercept. In this study, 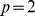 and 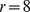.

This study assumes that the dependent variable 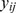 is Bernoulli distributed, i.e 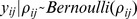 with an unknown 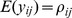, being related to the independent variables as follows:
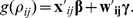



In this equation, 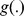 is a logit link function, 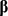 is a 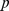 dimensional vector of regression coefficients for the continuous independent variables, and 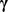 is a 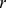
dimensional vector of regression coefficients for the categorical independent variables.

In order to cater for both the nonlinear effects of the continuous covariates and the spatial autocorrelation in the data, a semi-parametric model utilizing the penalized regression spline approach and convolution model was employed.

The penalized regression spline approach relaxed the highly restrictive linear predictor by a more flexible semi-parametric predictor, defined as:
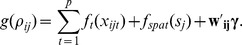



Here, 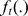 is a nonlinear twice differentiable smooth function for the continuous covariates and 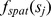 is a factor that caters for the spatial effects of each county. This study considers a convolution approach to these spatial effects, which assumes that the spatial effect can be decomposed in to two pure components, spatially structured and spatially unstructured, i.e 
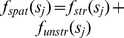
.

The final model can be expressed as:
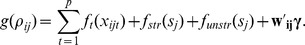



### Estimation of parameters

A full Bayesian approach in estimation was used in this study. Prior distributions were assigned to all the parameters as discussed in the following subsections.

### Smooth function

There exists a myriad of methods for estimating the smooth functions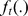. Several authors have given a thorough review of these methods [20]–[21]. Of particular importance to this current study is the use of penalized regression splines, which was proposed by Eilers and Marx [22]. In this method, the assumption is that the effect of the continuous covariates can be approximated by a polynomial spline. In particular, they assume that the smooth function 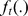 can be approximated by a spline of degree 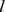 with 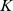 equally spaced knots, 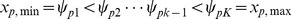, yielding, 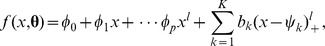
where, 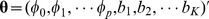 and 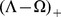 is equal to 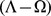 if 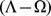 is positive and zero otherwise.

Following Caroll and Rupert [23], this study considers 20 knots to ensure enough flexibility and takes the 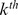 knot to be defined as the sample quantile of the continuous independent variable obtained with a probability equal to 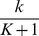. Furthermore, this study utilizes a quadratic spline, hence degree 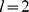.

To avoid getting a smooth function which “wiggles” too much, a roughness penalty, suggested by Green and Silverman [24], given by
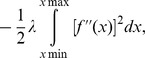
is imposed in the log- likelihood yielding the penalized log-likelihood function given by
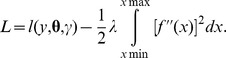



The balance between flexibility and smoothness is dictated by the positive parameter 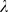.

### Spatial components priors

For the prior distribution of the spatially structured effects, this study uses the nearest neighbour Gaussian Markov random field which is specified as follows:
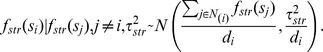



Here, 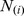
and 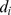 are, respectively, the set and number of neighbours of region 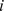. The neighbourhood can be defined in terms of whether two regions share a border or not. If the two regions share a border then they are neighbours, otherwise they are not. This leads to the intrinsic conditional autoregressive (ICAR) prior distribution [25], [26].

The unstructured spatial effect were assumed to have a Gaussian prior distribution, that is 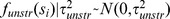.

In addition, the variance hyperparameters, 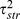 and 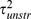 were assigned inverse gamma prior distributions as follows:
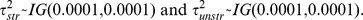



### Other prior distributions

For the fixed effects coefficients, the following prior distributions were assumed:
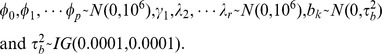



### Posterior distribution

Posterior distribution refers to the distribution of the parameters after observing the data. It is obtained by updating the prior distribution with observed data. A full Bayesian inference gets its estimates by sampling from this posterior distribution. In reality, the posterior distribution is usually of high dimension and analytically intractable. This is aggravated by the heavy integration required when performing analytical methods. Markov chain Monte Carlo (McMC) methods is a class of techniques used to overcome this problem. It allows for direct sampling from this posterior distribution repeatedly and estimates are calculated from these samples using simple data summaries such as mean and median.

Assuming conditional independence, the posterior distribution of the parameters for the Bernoulli model is given by
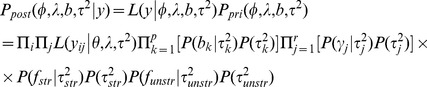



All the analyses in this study were carried out using WinBUGS 14 [27]. For each model, 40,000 Markov chain Monte Carlo (McMC) iterations were ran, with the initial 10,000 discarded to cater for the burn-in period and thereafter keeping every tenth sample value. The 3,000 iterations left were used for assessing convergence of the McMC and parameter estimation. We assessed McMC convergence of all models parameters by checking trace plots and autocorrelation plots of the McMC output [28]. Text S1 presents the WinBUGS code used during the analysis.

#### Model diagnostics

The models were compared using the Deviance Information Criterion (DIC) as suggested by Spiegelhalter et al. [29]. The best fitting model is one with the smallest DIC. DIC value is given by 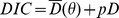, in which 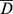 is the posterior mean of the deviance that measures the goodness of fit, and 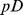 gives the effective number of parameters in the model which penalizes for complexity of the model. In this criterion, low vales of 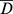 indicate a better fit while small values of 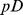 indicate a parsimonious model.

When comparing two models, how big the difference between the DIC values of the two models need to be so as to declare that one model is better than the other is not clear cut. However, several authors have stated that a difference in DIC of 3 between two models cannot be distinguished while a difference between 3 and 7 can be weakly differentiated [26], [29].

### Application/Data Analysis

The following set of models were investigated in order to understand the effect of the observed covariates and unobserved effects on the distribution of HIV in Kenya based on the male data:
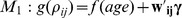


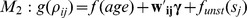


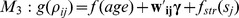


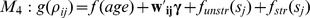



Model 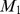 is a model of fixed categorical covariate and one continuous covariate, age, modelled with a non-linear smooth function and linear effects of the categorical covariates, place of residence, education level, marital status, age at first sex, perceived risk of HIV, circumcision status, if had STI in the last 12 months and if ever used a condom. The choice of modelling age with non-linear smoothing prior is supported by results from [14], [17]. This model does not take into account the spatially structured and spatially unstructured random effects.

Model 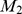 is an additive model that assumes a nonlinear function of the covariate age, linear effects of categorical covariates (listed in model 1 above) and spatially unstructured random effects which cover the unobserved covariates that are inherent within the counties.

The third model, 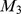, examines the effect of nonlinear covariate age, linear effects of categorical covariates and spatially structured random effect which accounts for any unobserved covariates that vary spatially among the counties.

The final model 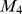 examines the effects of nonlinear effects of age, linear effects of categorical covariates and a convolution of spatially structured and spatially unstructured random effects. Table 2 summarizes the structure of nesting for the four models, under study, in order of complexity. The adjusted odds ratios, were reported, for these multivariate models. The adjustment is to cater for confounding between the variables.

**Table 2:** Nesting nature of the models under study.

Model	Nonlinear effect of age	Linear effects of categorical covariates	Spatially unstructured random effects	Spatially structured random effects
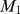	**√**	**√**	**-**	**-**
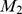	**√**	**√**	**√**	**-**
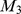	**√**	**√**	**-**	**√**
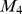	**√**	**√**	**√**	**√**

## Results

### Model assessment and comparison

Table 3 presents model diagnostics for all the fitted models. Model with a small DIC value provides a better fit. Comparing the goodness of fit and complexity of the models, model 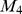 is the preferred model. In what follows, we only present the results based on this best fitting model (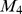).

**Table 3:** Models comparison.

	MODELS			
fit	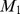	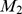	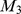	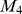
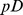	22.20	29.56	33.01	36.303
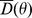	1393.89	1382.38	1377.86	1364.02
DIC	1416.09	1411.94	1410.87	1400.32

### Fixed effects

Table 4 gives the posterior estimates of the Odds ratios (OR) and their corresponding 95% credible intervals (CI) for the categorical covariates that were assumed to have a linear effect of HIV, based on the best fitting model 4. The following covariates were found to be significantly associated with HIV infection in this model: place of residence, education level, circumcision status, if the individual had an STI in the last 12 months and if the man has ever used a condom.

**Table 4:** Parameter estimates based on the best fitting model 4.

Fixed effects	Adjusted OR	95% CI for OR
**Demographic characteristics**		
Place of residence (*ref Rural*)	1	
*Urban*	1.88	(1.196,2.831)
**Social characteristics**		
Education level (*ref None*)	1	
*Primary*	1.248	(0.829,1.791)
*Secondary*	0.726	(0.449,1.096)
*Higher*	0.463	(0.232,0.815)
Marital status (*ref Married, 1partner*)	1	
*Married, +2partner*	1.125	(0.633,1.818)
*Divorced/Separated*	1.224	(0.461,2.459)
*Widowed*	4.548	(0.946,12.25)
*Never Married*	2.209	(1.062,3.952)
Age at first sex (*ref Never had sex*)	1	
*under 11*	2.690	(0.000,21.630)
*between 12–14*	5.525	(0.001,40.850)
*between 15–17*	4.383	(0.001,31.380)
*over 18*	3.404	(0.001,24.650)
Perceived risk of HIV (*ref No risk*)	1	
*Small Risk*	0.632	(0.282,1.202)
*Moderate Risk*	0.629	(0.308,1.172)
*Great Risk*	0.996	(0.456,1.937)
**Biological characteristics**		
Circumcision status (*ref Circumcised*)	1	
*Not circumcised*	4.422	(2.794,6.537)
Had STI in the last 12 months (*ref No*)	1	
*Yes*	2.946	(1.428,5.449)
**Behaviorial characteristics**		
Ever used Condom (*ref No*)	1	
*Yes*	1.506	(1.071,2.09)
**Random effects**	**Estimate**	**95% CI**
Spatially unstructured ( 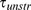 )	0.100	(0.000,0.435)
Spatially structured ( 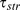 )	0.169	(0.001,0.895)
Spline coefficients ( 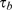 )	2947	(950.6,6789)

HIV infection is negatively related to education. The likelihood of HIV infection was higher for those men with primary education as compared to those with no education, albeit, this was not significant as indicated by the odds ratio and its corresponding credible interval (OR: 1.25, 95% CI: 0.83 to 1.79). The chance of HIV infection was lower for men with secondary education compared to those with no education, although this difference was not significant (OR: 0.73, 95% CI: 0.45 to 1.10). The likelihood of HIV infection was lowest in men with higher education (OR: 0.46, 95% CI: 0.23 to 0.82). Circumcision was found to be significantly associated with HIV infection. The odds of an uncircumcised man to be infected with HIV were 4.42 times the odds of a circumcised man (OR: 4.42, 95% CI: 2.79 to 6.54). Place of residence (Urban/Rural) was also found to be associated with HIV infection among men. The odds of HIV infection among men staying in urban areas was 1.88 times higher than that of men living in the rural areas (OR: 1.88, 95% CI: 1.20 to 2.83). Individuals who had an STI in the last 12 months were found to be 2.95 times more likely to be HIV positive (OR: 2.95, 95% CI: 1.43 to 5.45). Men who have ever used condoms were found to have an elevated likelihood of being infected by HIV (OR: 1.51, 95% CI: 1.07 to 2.09).

### Nonlinear effects of age

**Figure 1:**
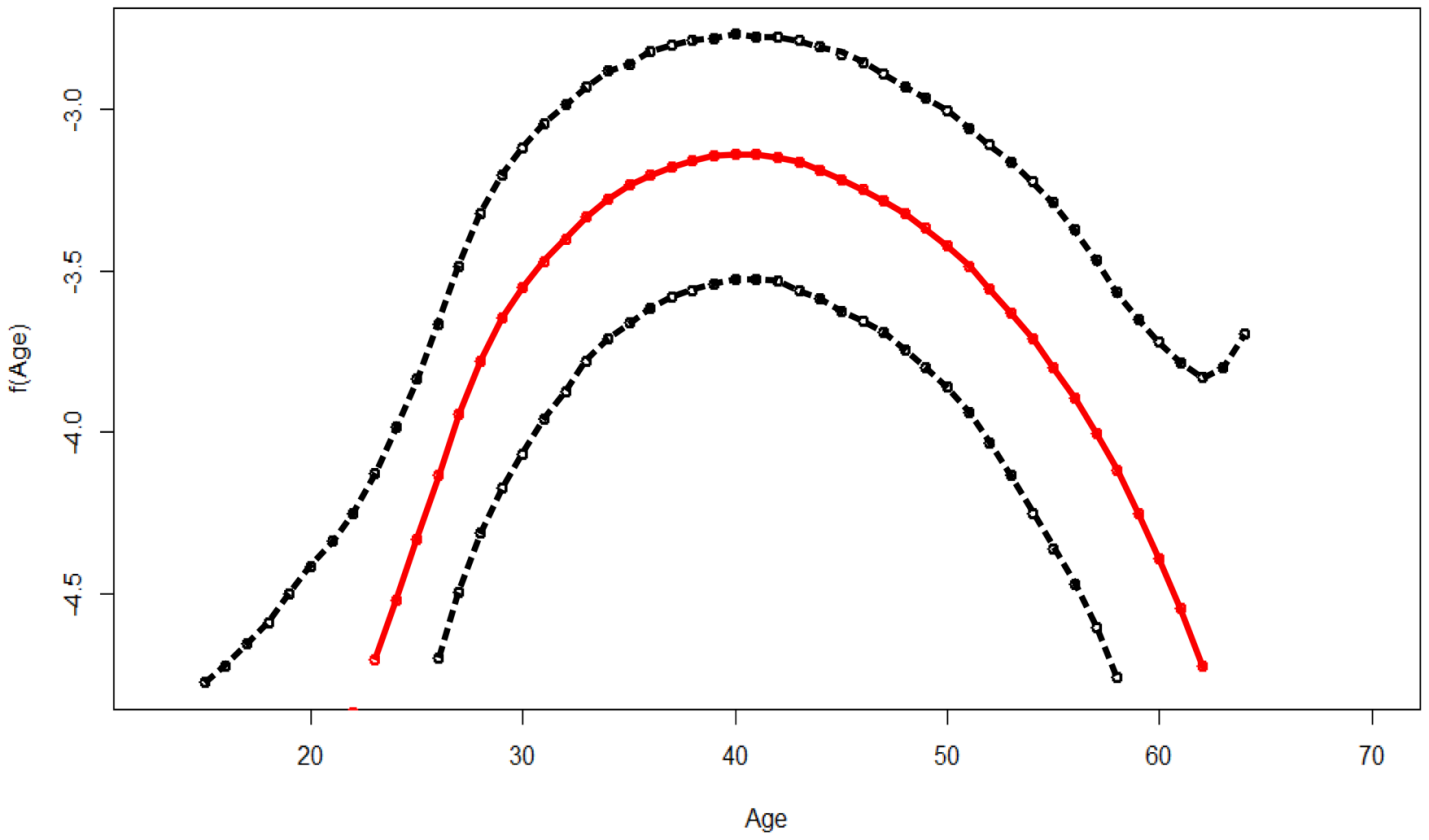
Estimated mean of the Nonlinear effect of age (in red) and the corresponding 95% credible interval (dotted black lines).

Figure 1 shows the nonlinear association between of age of an individual and the HIV infection. The figure gives the posterior mean of the smooth function and its corresponding 95% CI. It is evident from the figure that the effect of age on HIV infection is nonlinear, and an assumption of a linear effect would have led to specious results and subsequently wrong interpretations. The likelihood of HIV infection is seen to increase with age up to an optimum age of approximately 40 years then starts declining with increase with age.

### Spatial effects

**Figure 2:**
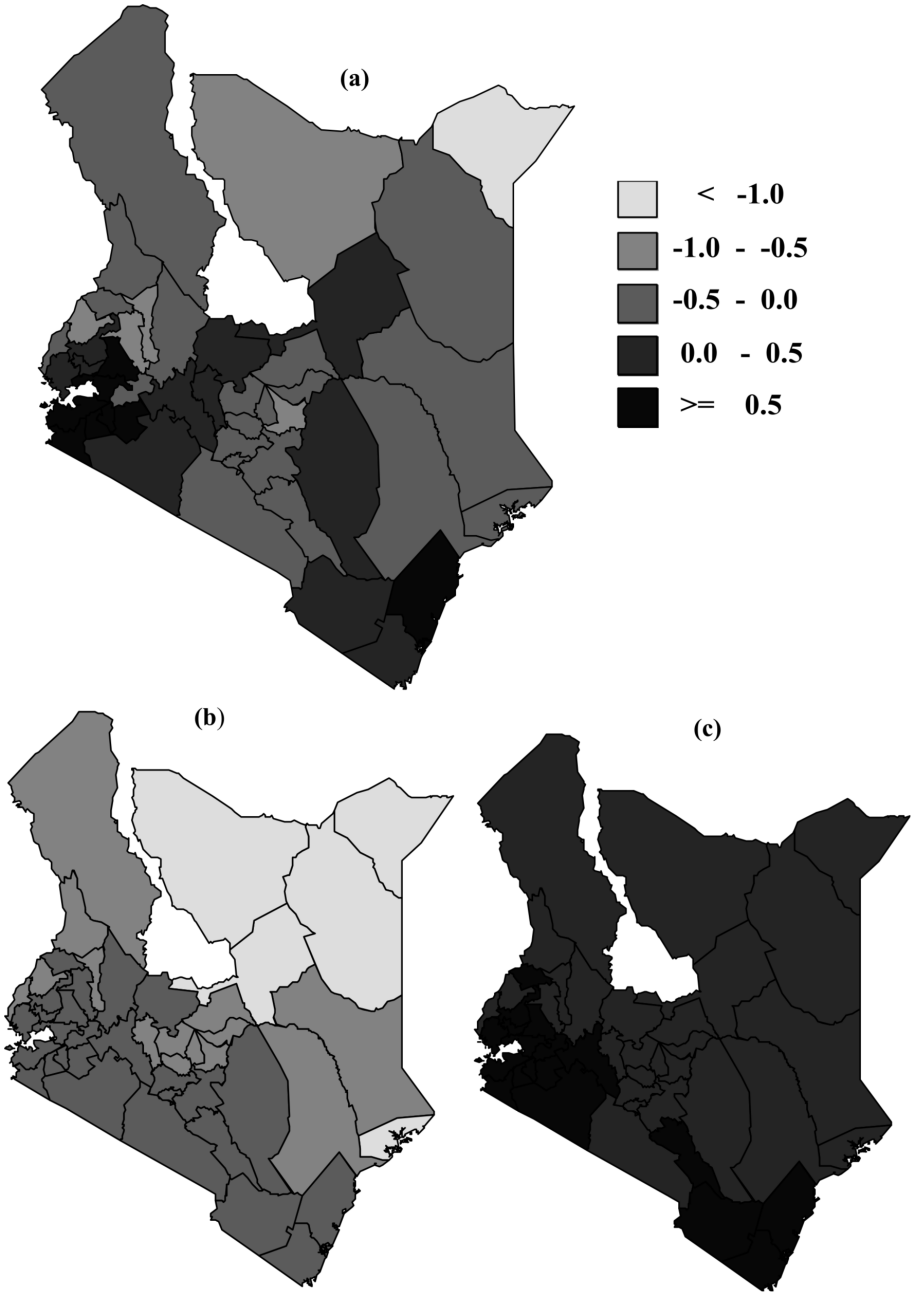
Residual spatial effect of county. Shown are the posterior means (a) and the corresponding lower (b) and upper (c) 95% credible limits.

**Figure 3:**
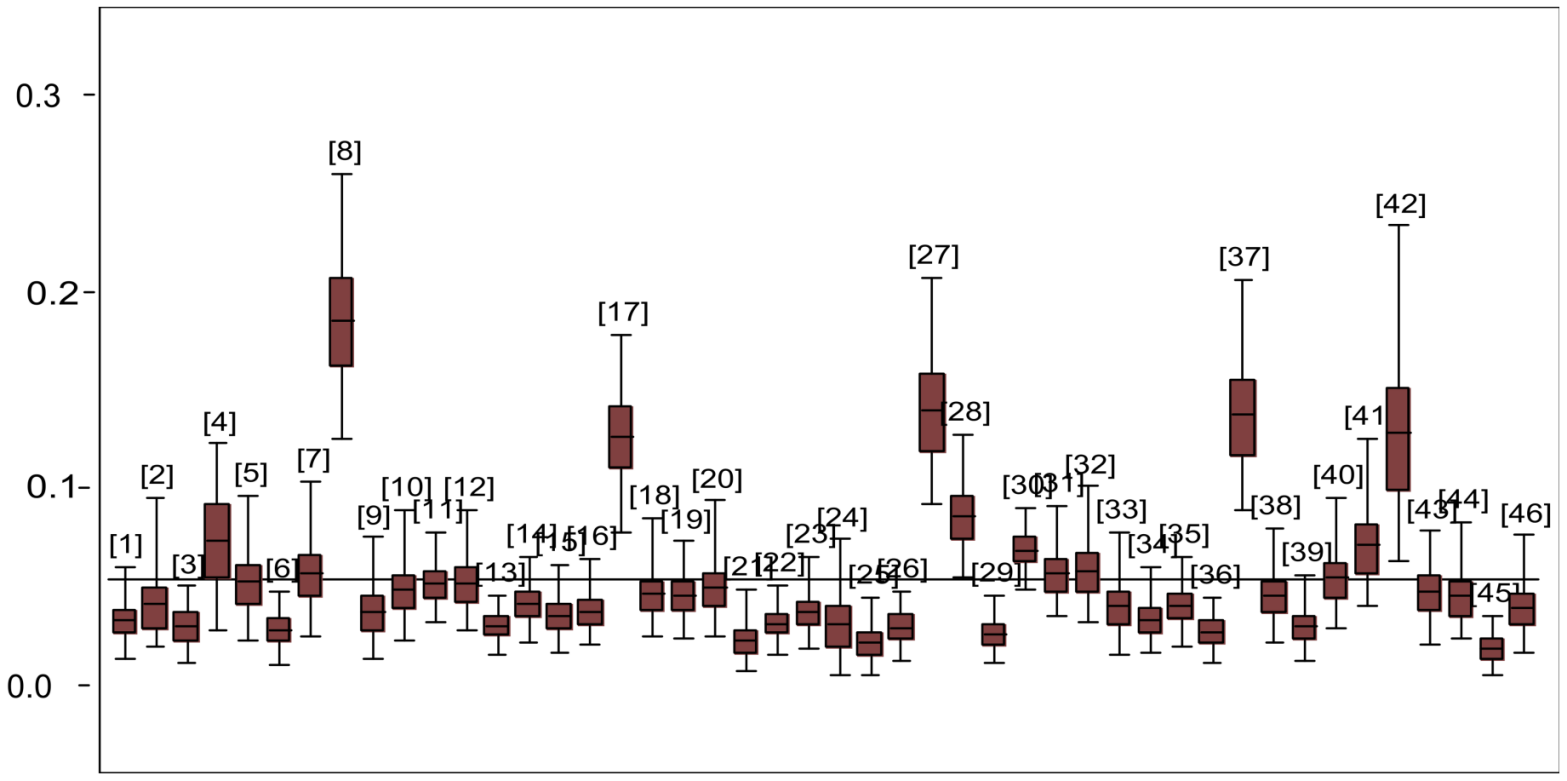
Box plot of HIV prevalence estimates by county. The numbers represent counties (See **table 5** for the county numbering codes).

We examined the spatial effects based on the best fitting model 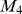. The spatial effect with the corresponding 95% CI is given in Figure 2. From the figure, counties with light grey colour show low association with HIV infection while black shaded counties have high association with HIV infection prevalence. There is clear evidence of spatial variation of HIV prevalence. From the maps, counties in the Western part, around Lake Victoria region, show high spatial variation. Very high HIV prevalence (>10%) was recorded in the following counties: Homabay, Migori, Siaya, Turkana, and Kisumu. Figure 3 gives a box-plot of HIV prevalence by counties (the counties are numbered from 1 to 46, see Table 5 for corresponding county names) while Table 5 gives the exact estimates.

**Table 5:** Estimated HIV prevalence by county and corresponding 95% Credible interval.

Code	County	Prevalence (95% CI)
1	Baringo	0.033(0.014,0.061)
2	Bomet	0.042(0.02,0.096)
3	Bungoma	0.03(0.012,0.051)
4	Busia	0.074(0.029,0.123)
5	Elgeyo Marakwet	0.052(0.023,0.095)
6	Embu	0.028(0.011,0.048)
7	Garissa	0.058(0.026,0.105)
8	Homa Bay	0.186(0.127,0.261)
9	Isiolo	0.038(0.014,0.074)
10	Kajiado	0.049(0.023,0.091)
11	Kakamega	0.052(0.033,0.078)
12	Kericho	0.052(0.028,0.09)
13	Kiambu	0.03(0.016,0.046)
14	Kilifi	0.041(0.022,0.066)
15	Kirinyaga	0.036(0.017,0.061)
16	Kisii	0.038(0.021,0.064)
17	Kisumu	0.126(0.076,0.177)
18	Kitui	0.046(0.025,0.083)
19	Kwale	0.046(0.024,0.074)
20	Laikipia	0.05(0.024,0.097)
21	Lamu	0.023(0.007,0.049)
22	Machakos	0.031(0.016,0.051)
23	Makueni	0.038(0.019,0.067)
24	Mandera	0.032(0.005,0.073)
25	Marsabit	0.021(0.005,0.043)
26	Meru	0.029(0.013,0.048)
27	Migori	0.14(0.093,0.204)
28	Mombasa	0.086(0.054,0.125)
29	Muranga	0.027(0.011,0.046)
30	Nairobi	0.069(0.05,0.09)
31	Nakuru	0.057(0.037,0.092)
32	Nandi	0.059(0.033,0.101)
33	Narok	0.04(0.016,0.08)
34	Nyamira	0.033(0.016,0.06)
35	Nyandarua	0.04(0.02,0.065)
36	Nyeri	0.027(0.011,0.044)
37	Siaya	0.137(0.089,0.204)
38	Taita Taveta	0.046(0.022,0.079)
39	Tana River	0.03(0.012,0.057)
40	Tharaka-Nithi	0.054(0.028,0.097)
41	Trans Nzoia	0.071(0.04,0.127)
42	Turkana	0.129(0.064,0.238)
43	Uasin Gishu	0.048(0.02,0.078)
44	Vihiga	0.045(0.024,0.081)
45	Wajir	0.019(0.005,0.036)
46	West Pokot	0.039(0.017,0.076)

The structured spatial effects are dominant over the spatially unstructured random effects as shown by the ratio of variance components, calculated by 
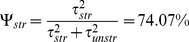
 (Table 4).

## Discussion

This study utilizes Bayesian techniques to analyze regional variation and risk factors of HIV infection. The study develops and uses Bayesian semi-parametric regression models to help assess factors associated with HIV infection. We build on existing contributions by Eilers and Marx [22] and Caroll and Rupert [23]. The models used in this study are based on the penalized regression splines, in a semi-parametric modeling paradigm, with allowance for spatial variation in the response variable. Many situations arise where the relationship between the function of the response variable and covariates is not pigeonholed linearly [21]. Semi-parametric models are a class of models which borrows strength from both parametric and non-parametric models. Subsequently, it can be viewed as enriching standard parametric models by exploring the non-parametric domain while still keeping intact the linear structure [30]. Furthermore, the regularly encountered assumption of fully parametric linear predictor for evaluating the effect of covariates on the dependent variable is usually restrictive, unrealistic and can lead to specious conclusions. The spatially structured effects in the model are modeled using a Gaussian Markov Random Field (GMRF) while the spatially unstructured random effects are modeled using a zero mean Gaussian process [25], [26]. Due to the high complexity and intractability owing to this intricacy, the maximum likelihood approaches are not viable in the estimation of these models. Consequently, the Bayesian inference, utilizing McMC techniques is highly favoured.

In this study, we found that the male circumcision reduces the risk of HIV infection, precisely, uncircumcised men were more likely to be HIV positive than circumcised men. This finding is supported by previous studies and therefore adds to the large body of research indicating that circumcision lowers the risk of HIV infection [4]–[6]. The Ministry of Health in Kenya currently runs an aggressive Voluntary Medical Male Circumcision (VMMC) in the country [31]–[32]. With respect to this finding, there will be hope for decline in HIV prevalence due to this campaign.

Place of residence proved to have a significant relationship to HIV infection among men when controlled for other covariates. Men in urban areas were more likely to be HIV positive as compared to men in the rural areas. The effect of place of residence on HIV infection has been reported in many studies but with mixed conclusions [14], [33]. This finding could be used to inform tailormade HIV campaign strategies depending on place of residence.

Another finding from this study is that the likelihood of HIV infection was lowest among men with higher education. Other studies have also shown similar result [14]. The government's introduction of free primary education and subsidized secondary education is hoped to increase the number of young people attaining higher level of education [34]. This is envisaged to increase awareness of this pandemic and further reduce its spread.

It was also found in this study that men who had an STI in the last 12 months were more likely to be HIV positive. In terms of condom usage, men who have ever used a condom were more likely to be infected by HIV. This is an unexpected finding. This finding may be attributed to two possible reasons. Firstly, some men who use condom, with their spouses, will later on end up stopping to use a condom by assuming that they “know” one another properly and trust each other. Subsequently having unprotected sex. Secondly, the way this question was captured in the study, wasn't useful. A better way was to capture consistent use of condom. If this could have been captured then, maybe, opposite and expected results could have been realized.

The nonlinear effect of age on HIV infection was evident from the analysis. The relationship between HIV infection and age has an inverted “U” shape. The likelihood of HIV infection increase with age and reaches a maximum at the age of around 40, then takes a nosedive. This result is in tandem with other bodies of research [14], [17].

Spatial effects in the model acts as surrogate of the unobserved variables. Identification of high prevalence areas can provide insights for designing intervention and campaign programmes that are tailor-made to those regions, hence increasing the impact of the initiative. There was evidence of spatial variation of HIV infection among the counties, with highest prevalence rates being reported on the Western part of the country, around Lake Victoria region.

Vis-a-vis classical regression modeling frameworks, this study uses an approach that allows for flexible and realistic models to be implemented, thereby making it possible to establish and test epidemiological hypotheses. Availability of freely available software like R and WinBUGS makes implementation of these complex models easier and accessible to practitioners in medical, environmental and any other relevant field.

A major limitation of our analysis is that the data used for county estimation was collected when the country was still based on the old administrative units (provinces), the data was not powered to carry out estimation at these new administrative units. The study rides on the advantage that these new administrative units called counties were formed by combining several districts together. This made it easy for the county where an individual belongs to be allocated easily since each district belongs to only one county. Also, in the data, the way some variables were captured was not usefull; ever using a condom should be replaced with consistent use of condom. In terms of methodology, the knots used in the penalized spline regression were assumed to be fixed and were calculated as quantiles from the continuous variable age. A more flexible analysis can allow the knots to be data driven [35].

Despite the limitations highlighted above, the models introduced in this study can be replicated in other countries with similar data.

## Supporting Information

Text S1 **WinBUGS code used in the analysis.**
(DOCX)
