# Supplementary material for: Bayesian Spatial Semi-Parametric Modeling of HIV Variation in Kenya
Source: PLoS One. 2014 Jul 25;9(7):e103299. doi: 10.1371/journal.pone.0103299 (PMC4111556; doi:10.1371/journal.pone.0103299)
Supplement: Text S1 — WinBUGS code used in the analysis. (DOCX) [file pone.0103299.s001.docx]

**WINBUGS CODES IN** “**BAYESIAN SPATIAL SEMI-PARAMETRIC MODELING OF HIV VARIATION IN KENYA”**

##############fixed effect with spline###############

model

{

#likelihood

for(i in 1: N)

{

for(l in 1:degree+1)

{

X[i,l]<-pow(Age[i],l-1)

}

}

for(i in 1: N)

{

for(k in 1:20)

{

u[i,k]<-(Age[i]-knot[k])*step(Age[i]-knot[k])

Z[i,k]<-pow(u[i,k],degree)

}

}

for(i in 1: N)

{

###Definition of Variables##

#########Ever used condom###########

D.Ever_used_condom[i]<-equals(Ever_used_condom[i],1)

###################

##########STI##########

####STI=1 No STI=2#########

D.STI [i]<-equals(STI[i],1)

########CIRCUMCISED#############

###circumcised=1, notcircumcised=2##

D.Circumcised[i]<-equals(Circumcised[i],2)

#########URBAN##################

###urban=1, rural=0##

D.Urban[i]<-equals(Urban[i],1)

#####EDUCATION############

###None=0,primary=1,secondary=2,higher=3##

D.education2[i]<-equals(education[i],1)

D.education3[i]<-equals(education[i],2)

D.education4[i]<-equals(education[i],3)

#######MARITAL STATUS###########

#married 1 partner =1, married 2+ partners =2.... ###

D.MaritalStatus2[i]<-equals(MaritalStatus[i],2)

D.MaritalStatus3[i]<-equals(MaritalStatus[i],3)

D.MaritalStatus4[i]<-equals(MaritalStatus[i],4)

D.MaritalStatus5[i]<-equals(MaritalStatus[i],5)

########AGE AT FIRST SEX########

######Never had sex=0, under 11=1, ####

D.age_first_sex2[i]<-equals( age_first_sex[i],1)

D.age_first_sex3[i]<-equals( age_first_sex[i],2)

D.age_first_sex4[i]<-equals( age_first_sex[i],3)

D.age_first_sex5[i]<-equals( age_first_sex[i],4)

########Perceived RISK##########

### no risk =0####

D.perceived_Risk2[i]<-equals(perceived_Risk[i],1)

D.perceived_Risk3[i]<-equals(perceived_Risk[i],2)

D.perceived_Risk4[i]<-equals(perceived_Risk[i],3)

#for HIV

hiv[i]~dbern(p1[i])

p1[i]<-min(1,max(0,PHIV[i]))

logit(PHIV[i])<- fixedvars1[i]+fixedvars2[i]+fixedvars3[i]+fixedvars4[i]+fixedvars5[i]+spline[i]

fixedvars1[i]<-edu1[2]*D.education2[i]+edu1[3]*D.education3[i]+edu1[4]*D.education4[i]

fixedvars2[i]<-Circum1*D.Circumcised[i]+Urb1*D.Urban[i]+STI1*D.STI[i]

fixedvars3[i]<-Ever*D.Ever_used_condom[i]+marital[2]*D.MaritalStatus2[i]+marital[3]*D.MaritalStatus3[i]+marital[4]*D.MaritalStatus4[i]+marital[5]*D.MaritalStatus5[i]

fixedvars4[i]<-Fsex[2]*D.age_first_sex2[i]+Fsex[3]*D.age_first_sex3[i]+Fsex[4]*D.age_first_sex4[i]+Fsex[5]*D.age_first_sex5[i]

fixedvars5[i]<-perceive[2]*D.perceived_Risk2[i]+perceive[3]*D.perceived_Risk3[i]+perceive[4]*D.perceived_Risk4[i]

spline[i]<-inprod(b[ ], Z[i, ])+inprod(beta[ ], X[i, ])

}

#nuissance parameters set to zero..to cater for reference levels

edu1[1]<-0

marital[1]<-0

Fsex[1]<-0

perceive[1]<-0

#priors

Circum1~dnorm(0.0,0.000001)

Urb1~dnorm(0.0,0.000001)

STI1~dnorm(0.0,0.000001)

Ever~dnorm(0.0,0.000001)

# Educationcoefficients

for(j in 2: 4)

{

edu1[j]~dnorm(0.0,0.000001)

}

# Marital coefficients

for(j in 2: 5)

{

marital[j]~dnorm(0.0,0.000001)

}

#Age at first sex coefficients

for(j in 2: 5)

{

Fsex[j]~dnorm(0.0,0.000001)

}

#Perception coefficients

for(j in 2: 4)

{

perceive[j]~dnorm(0.0,0.000001)

}

#fixedvars coefficients

for(l in 1:degree+1)

{

beta[l]~dnorm(0,0.000001 )

}

#priorsplines

for(k in 1:20)

{

b[k]~dnorm(0,taub )

}

taub~dgamma(0.001,0.001)

#ODDS ratios

#Education coefficients

for(j in 1: 4)

{

ORedu1[j]<-exp(edu1[j])

}

for(j in 1: 5)

{

ORmarital[j]<-exp(marital[j])

}

for(j in 1: 5)

{

ORFsex[j]<-exp(Fsex[j])

}

for(j in 1: 4)

{

ORperceive[j]<-exp(perceive[j])

}

ORCircum1<-exp(Circum1)

ORUrb1<-exp(Urb1)

OREver<-exp(Ever)

ORSTI<-exp(STI1)

for(i in 1: N)

{

for(j in 1: 46)

{

PH[j,i]<-(PHIV[i])*(equals(county[i],j))

}

}

for(j in 1: 46)

{

for(i in 1: N)

{

count[j,i]<-equals(county[i],j)

}

number[j]<-sum(count[j,])

PCHV[j]<-sum(PH[j,])/number[j]

}

}

#DATA latest

#INITIALS

list(taub=0.001, beta=c(0,0,0),Circum1=0,Urb1=0,STI1=0, Ever=0, edu1=c(NA,0,0,0),marital=c(NA,0,0,0,0),Fsex=c(NA,0,0,0,0),perceive=c(NA,0,0,0),b=c(0,0,0,0,0,0,0,0,0,0,0,0,0,0,0,0,0,0,0,0))

######Spatially unstructured random effect with spline####

model

{

#likelihood

for(i in 1: N)

{

for(l in 1:degree+1)

{

X[i,l]<-pow(Age[i],l-1)

}

}

for(i in 1: N)

{

for(k in 1:20)

{

u[i,k]<-(Age[i]-knot[k])*step(Age[i]-knot[k])

Z[i,k]<-pow(u[i,k],degree)

}

}

for(i in 1: N)

{

###Definition of Variables##

#########Ever used condom###########

D.Ever_used_condom[i]<-equals(Ever_used_condom[i],1)

###################

##########STI##########

####STI=1 No STI=2#########

D.STI [i]<-equals(STI[i],1)

########CIRCUMCISED#############

###circumcised=1, notcircumcised=2##

D.Circumcised[i]<-equals(Circumcised[i],2)

#########URBAN##################

###urban=1, rural=0##

D.Urban[i]<-equals(Urban[i],1)

#####EDUCATION############

###None=0,primary=1,secondary=2,higher=3##

D.education2[i]<-equals(education[i],1)

D.education3[i]<-equals(education[i],2)

D.education4[i]<-equals(education[i],3)

#######MARITAL STATUS###########

#married 1 partner =1, married 2+ partners =2.... ###

D.MaritalStatus2[i]<-equals(MaritalStatus[i],2)

D.MaritalStatus3[i]<-equals(MaritalStatus[i],3)

D.MaritalStatus4[i]<-equals(MaritalStatus[i],4)

D.MaritalStatus5[i]<-equals(MaritalStatus[i],5)

########AGE AT FIRST SEX########

######Never had sex=0, under 11=1, ####

D.age_first_sex2[i]<-equals( age_first_sex[i],1)

D.age_first_sex3[i]<-equals( age_first_sex[i],2)

D.age_first_sex4[i]<-equals( age_first_sex[i],3)

D.age_first_sex5[i]<-equals( age_first_sex[i],4)

########Perceived RISK##########

### no risk =0####

D.perceived_Risk2[i]<-equals(perceived_Risk[i],1)

D.perceived_Risk3[i]<-equals(perceived_Risk[i],2)

D.perceived_Risk4[i]<-equals(perceived_Risk[i],3)

#for HIV

hiv[i]~dbern(p1[i])

p1[i]<-min(1,max(0,PHIV[i]))

logit(PHIV[i])<- fixedvars1[i]+fixedvars2[i]+fixedvars3[i]+fixedvars4[i]+fixedvars5[i]+spline[i]

fixedvars1[i]<-edu1[2]*D.education2[i]+edu1[3]*D.education3[i]+edu1[4]*D.education4[i]

fixedvars2[i]<-Circum1*D.Circumcised[i]+Urb1*D.Urban[i]+STI1*D.STI[i]

fixedvars3[i]<-Ever*D.Ever_used_condom[i]+marital[2]*D.MaritalStatus2[i]+marital[3]*D.MaritalStatus3[i]+marital[4]*D.MaritalStatus4[i]+marital[5]*D.MaritalStatus5[i]

fixedvars4[i]<-Fsex[2]*D.age_first_sex2[i]+Fsex[3]*D.age_first_sex3[i]+Fsex[4]*D.age_first_sex4[i]+Fsex[5]*D.age_first_sex5[i]

fixedvars5[i]<-perceive[2]*D.perceived_Risk2[i]+perceive[3]*D.perceived_Risk3[i]+perceive[4]*D.perceived_Risk4[i]+v[county[i]]

spline[i]<-inprod(b[ ], Z[i, ])+inprod(beta[ ], X[i, ])

}

sigma.v<-(1/tau.v)

tau.v~dgamma(0.1,0.0001)

for(j in 1: 46)

{

v[j]~dnorm(0,tau.v)

}

#nuissance parameters set to zero..to cater for reference levels

edu1[1]<-0

marital[1]<-0

Fsex[1]<-0

perceive[1]<-0

#priors

Circum1~dnorm(0.0,0.000001)

Urb1~dnorm(0.0,0.000001)

STI1~dnorm(0.0,0.000001)

Ever~dnorm(0.0,0.000001)

# Educationcoefficients

for(j in 2: 4)

{

edu1[j]~dnorm(0.0,0.000001)

}

# Marital coefficients

for(j in 2: 5)

{

marital[j]~dnorm(0.0,0.000001)

}

#Age at first sex coefficients

for(j in 2: 5)

{

Fsex[j]~dnorm(0.0,0.000001)

}

#Perception coefficients

for(j in 2: 4)

{

perceive[j]~dnorm(0.0,0.000001)

}

#fixedvars coefficients

for(l in 1:degree+1)

{

beta[l]~dnorm(0,0.000001 )

}

#priorsplines

for(k in 1:20)

{

b[k]~dnorm(0,taub )

}

taub~dgamma(0.001,0.001)

#ODDS ratios

#Education coefficients

for(j in 1: 4)

{

ORedu1[j]<-exp(edu1[j])

}

for(j in 1: 5)

{

ORmarital[j]<-exp(marital[j])

}

for(j in 1: 5)

{

ORFsex[j]<-exp(Fsex[j])

}

for(j in 1: 4)

{

ORperceive[j]<-exp(perceive[j])

}

ORCircum1<-exp(Circum1)

ORUrb1<-exp(Urb1)

OREver<-exp(Ever)

ORSTI<-exp(STI1)

for(i in 1: N)

{

for(j in 1: 46)

{

PH[j,i]<-(PHIV[i])*(equals(county[i],j))

}

}

for(j in 1: 46)

{

for(i in 1: N)

{

count[j,i]<-equals(county[i],j)

}

number[j]<-sum(count[j,])

PCHV[j]<-sum(PH[j,])/number[j]

}

}

#DATA latest

#INITIALS

list(taub=0.001, beta=c(0,0,0),Circum1=0,Urb1=0,STI1=0, Ever=0, edu1=c(NA,0,0,0),marital=c(NA,0,0,0,0),Fsex=c(NA,0,0,0,0),perceive=c(NA,0,0,0), b=c(0,0,0,0,0,0,0,0,0,0,0,0,0,0,0,0,0,0,0,0),tau.v=0.01,v=c(0, 0, 0, 0, 0, 0, 0, 0, 0, 0, 0, 0, 0, 0, 0, 0, 0, 0, 0, 0, 0, 0, 0, 0, 0, 0, 0, 0, 0, 0, 0, 0, 0, 0, 0, 0, 0, 0, 0, 0, 0, 0, 0, 0, 0 ,0))

#####Spatially structured random effect with spline#######

model

{

#likelihood

for(i in 1: N)

{

for(l in 1:degree+1)

{

X[i,l]<-pow(Age[i],l-1)

}

}

for(i in 1: N)

{

for(k in 1:20)

{

u[i,k]<-(Age[i]-knot[k])*step(Age[i]-knot[k])

Z[i,k]<-pow(u[i,k],degree)

}

}

for(i in 1: N)

{

###Definition of Variables##

#########Ever used condom###########

D.Ever_used_condom[i]<-equals(Ever_used_condom[i],1)

###################

##########STI##########

####STI=1 No STI=2#########

D.STI [i]<-equals(STI[i],1)

########CIRCUMCISED#############

###circumcised=1, notcircumcised=2##

D.Circumcised[i]<-equals(Circumcised[i],2)

#########URBAN##################

###urban=1, rural=0##

D.Urban[i]<-equals(Urban[i],1)

#####EDUCATION############

###None=0,primary=1,secondary=2,higher=3##

D.education2[i]<-equals(education[i],1)

D.education3[i]<-equals(education[i],2)

D.education4[i]<-equals(education[i],3)

#######MARITAL STATUS###########

#married 1 partner =1, married 2+ partners =2.... ###

D.MaritalStatus2[i]<-equals(MaritalStatus[i],2)

D.MaritalStatus3[i]<-equals(MaritalStatus[i],3)

D.MaritalStatus4[i]<-equals(MaritalStatus[i],4)

D.MaritalStatus5[i]<-equals(MaritalStatus[i],5)

########AGE AT FIRST SEX########

######Never had sex=0, under 11=1, ####

D.age_first_sex2[i]<-equals( age_first_sex[i],1)

D.age_first_sex3[i]<-equals( age_first_sex[i],2)

D.age_first_sex4[i]<-equals( age_first_sex[i],3)

D.age_first_sex5[i]<-equals( age_first_sex[i],4)

########Perceived RISK##########

### no risk =0####

D.perceived_Risk2[i]<-equals(perceived_Risk[i],1)

D.perceived_Risk3[i]<-equals(perceived_Risk[i],2)

D.perceived_Risk4[i]<-equals(perceived_Risk[i],3)

#for HIV

hiv[i]~dbern(p1[i])

p1[i]<-min(1,max(0,PHIV[i]))

logit(PHIV[i])<- fixedvars1[i]+fixedvars2[i]+fixedvars3[i]+fixedvars4[i]+fixedvars5[i]+spline[i]

fixedvars1[i]<-edu1[2]*D.education2[i]+edu1[3]*D.education3[i]+edu1[4]*D.education4[i]

fixedvars2[i]<-Circum1*D.Circumcised[i]+Urb1*D.Urban[i]+STI1*D.STI[i]

fixedvars3[i]<-Ever*D.Ever_used_condom[i]+marital[2]*D.MaritalStatus2[i]+marital[3]*D.MaritalStatus3[i]+marital[4]*D.MaritalStatus4[i]+marital[5]*D.MaritalStatus5[i]

fixedvars4[i]<-Fsex[2]*D.age_first_sex2[i]+Fsex[3]*D.age_first_sex3[i]+Fsex[4]*D.age_first_sex4[i]+Fsex[5]*D.age_first_sex5[i]

fixedvars5[i]<-perceive[2]*D.perceived_Risk2[i]+perceive[3]*D.perceived_Risk3[i]+perceive[4]*D.perceived_Risk4[i]+U[county[i]]

spline[i]<-inprod(b[ ], Z[i, ])+inprod(beta[ ], X[i, ])

}

#Spatiallly structured random effect prior

omega.spatial1 ~ dgamma(0.5, 0.0005)

omega.spatial1sq<-1/omega.spatial1

U[1 : 46] ~ car.normal(adj[], weights1[], num[], omega.spatial1)

for (k in 1:sumNumNeigh) {

weights1[k] <- 1

}

#nuissance parameters set to zero..to cater for reference levels

edu1[1]<-0

marital[1]<-0

Fsex[1]<-0

perceive[1]<-0

#priors

Circum1~dnorm(0.0,0.000001)

Urb1~dnorm(0.0,0.000001)

STI1~dnorm(0.0,0.000001)

Ever~dnorm(0.0,0.000001)

# Educationcoefficients

for(j in 2: 4)

{

edu1[j]~dnorm(0.0,0.000001)

}

# Marital coefficients

for(j in 2: 5)

{

marital[j]~dnorm(0.0,0.000001)

}

#Age at first sex coefficients

for(j in 2: 5)

{

Fsex[j]~dnorm(0.0,0.000001)

}

#Perception coefficients

for(j in 2: 4)

{

perceive[j]~dnorm(0.0,0.000001)

}

#fixedvars coefficients

for(l in 1:degree+1)

{

beta[l]~dnorm(0,0.000001 )

}

#priorsplines

for(k in 1:20)

{

b[k]~dnorm(0,taub )

}

taub~dgamma(0.001,0.001)

#ODDS ratios

#Education coefficients

for(j in 1: 4)

{

ORedu1[j]<-exp(edu1[j])

}

for(j in 1: 5)

{

ORmarital[j]<-exp(marital[j])

}

for(j in 1: 5)

{

ORFsex[j]<-exp(Fsex[j])

}

for(j in 1: 4)

{

ORperceive[j]<-exp(perceive[j])

}

ORCircum1<-exp(Circum1)

ORUrb1<-exp(Urb1)

OREver<-exp(Ever)

ORSTI<-exp(STI1)

for(i in 1: N)

{

for(j in 1: 46)

{

PH[j,i]<-(PHIV[i])*(equals(county[i],j))

}

}

for(j in 1: 46)

{

for(i in 1: N)

{

count[j,i]<-equals(county[i],j)

}

number[j]<-sum(count[j,])

PCHV[j]<-sum(PH[j,])/number[j]

}

}

#DATA latest

#INITIALS

list(taub=0.001, beta=c(0,0,0), Circum1=0,Urb1=0,STI1=0, Ever=0, edu1=c(NA,0,0,0),marital=c(NA,0,0,0,0),Fsex=c(NA,0,0,0,0),perceive=c(NA,0,0,0),b=c(0,0,0,0,0,0,0,0,0,0,0,0,0,0,0,0,0,0,0,0),omega.spatial1=0.01,U=c(0, 0, 0, 0, 0, 0, 0, 0, 0, 0, 0, 0, 0, 0, 0, 0, 0, 0, 0, 0, 0, 0, 0, 0, 0, 0, 0, 0, 0, 0, 0, 0, 0, 0, 0, 0, 0, 0, 0, 0, 0, 0, 0, 0, 0 ,0))

##############Convolution with spline###############

model

{

#likelihood

for(i in 1: N)

{

for(l in 1:degree+1)

{

X[i,l]<-pow(Age[i],l-1)

}

}

for(i in 1: N)

{

for(k in 1:20)

{

u[i,k]<-(Age[i]-knot[k])*step(Age[i]-knot[k])

Z[i,k]<-pow(u[i,k],degree)

}

}

for(i in 1: N)

{

###Definition of Variables##

#########Ever used condom###########

D.Ever_used_condom[i]<-equals(Ever_used_condom[i],1)

###################

##########STI##########

####STI=1 No STI=2#########

D.STI [i]<-equals(STI[i],1)

########CIRCUMCISED#############

###circumcised=1, notcircumcised=2##

D.Circumcised[i]<-equals(Circumcised[i],2)

#########URBAN##################

###urban=1, rural=0##

D.Urban[i]<-equals(Urban[i],1)

#####EDUCATION############

###None=0,primary=1,secondary=2,higher=3##

D.education2[i]<-equals(education[i],1)

D.education3[i]<-equals(education[i],2)

D.education4[i]<-equals(education[i],3)

#######MARITAL STATUS###########

#married 1 partner =1, married 2+ partners =2.... ###

D.MaritalStatus2[i]<-equals(MaritalStatus[i],2)

D.MaritalStatus3[i]<-equals(MaritalStatus[i],3)

D.MaritalStatus4[i]<-equals(MaritalStatus[i],4)

D.MaritalStatus5[i]<-equals(MaritalStatus[i],5)

########AGE AT FIRST SEX########

######Never had sex=0, under 11=1, ####

D.age_first_sex2[i]<-equals( age_first_sex[i],1)

D.age_first_sex3[i]<-equals( age_first_sex[i],2)

D.age_first_sex4[i]<-equals( age_first_sex[i],3)

D.age_first_sex5[i]<-equals( age_first_sex[i],4)

########Perceived RISK##########

### no risk =0####

D.perceived_Risk2[i]<-equals(perceived_Risk[i],1)

D.perceived_Risk3[i]<-equals(perceived_Risk[i],2)

D.perceived_Risk4[i]<-equals(perceived_Risk[i],3)

#for HIV

hiv[i]~dbern(p1[i])

p1[i]<-min(1,max(0,PHIV[i]))

logit(PHIV[i])<- fixedvars1[i]+fixedvars2[i]+fixedvars3[i]+fixedvars4[i]+fixedvars5[i]+spline[i]

fixedvars1[i]<-edu1[2]*D.education2[i]+edu1[3]*D.education3[i]+edu1[4]*D.education4[i]

fixedvars2[i]<-Circum1*D.Circumcised[i]+Urb1*D.Urban[i]+STI1*D.STI[i]

fixedvars3[i]<-Ever*D.Ever_used_condom[i]+marital[2]*D.MaritalStatus2[i]+marital[3]*D.MaritalStatus3[i]+marital[4]*D.MaritalStatus4[i]+marital[5]*D.MaritalStatus5[i]

fixedvars4[i]<-Fsex[2]*D.age_first_sex2[i]+Fsex[3]*D.age_first_sex3[i]+Fsex[4]*D.age_first_sex4[i]+Fsex[5]*D.age_first_sex5[i]

fixedvars5[i]<-perceive[2]*D.perceived_Risk2[i]+perceive[3]*D.perceived_Risk3[i]+perceive[4]*D.perceived_Risk4[i]+U[county[i]]+V[county[i]]

spline[i]<-inprod(b[ ], Z[i, ])+inprod(beta[ ], X[i, ])

}

#Spatiallly unstructured random effect prior

sigma.V<-(1/tau.V)

tau.V~dgamma(0.1,0.0001)

for(j in 1: 46)

{

V[j]~dnorm(0,tau.V)

}

#Spatiallly structured random effect prior

omega.spatial1 ~ dgamma(0.5, 0.0005)

omega.spatial1sq<-1/omega.spatial1

U[1 : 46] ~ car.normal(adj[], weights1[], num[], omega.spatial1)

for (k in 1:sumNumNeigh) {

weights1[k] <- 1

}

#nuissance parameters set to zero..to cater for reference levels

edu1[1]<-0

marital[1]<-0

Fsex[1]<-0

perceive[1]<-0

#priors

Circum1~dnorm(0.0,0.000001)

Urb1~dnorm(0.0,0.000001)

STI1~dnorm(0.0,0.000001)

Ever~dnorm(0.0,0.000001)

# Educationcoefficients

for(j in 2: 4)

{

edu1[j]~dnorm(0.0,0.000001)

}

# Marital coefficients

for(j in 2: 5)

{

marital[j]~dnorm(0.0,0.000001)

}

#Age at first sex coefficients

for(j in 2: 5)

{

Fsex[j]~dnorm(0.0,0.000001)

}

#Perception coefficients

for(j in 2: 4)

{

perceive[j]~dnorm(0.0,0.000001)

}

#fixedvars coefficients

for(l in 1:degree+1)

{

beta[l]~dnorm(0,0.000001 )

}

#priorsplines

for(k in 1:20)

{

b[k]~dnorm(0,taub )

}

taub~dgamma(0.001,0.001)

#ODDS ratios

#Education coefficients

for(j in 1: 4)

{

ORedu1[j]<-exp(edu1[j])

}

for(j in 1: 5)

{

ORmarital[j]<-exp(marital[j])

}

for(j in 1: 5)

{

ORFsex[j]<-exp(Fsex[j])

}

for(j in 1: 4)

{

ORperceive[j]<-exp(perceive[j])

}

ORCircum1<-exp(Circum1)

ORUrb1<-exp(Urb1)

OREver<-exp(Ever)

ORSTI<-exp(STI1)

for(i in 1: N)

{

for(j in 1: 46)

{

PH[j,i]<-(PHIV[i])*(equals(county[i],j))

}

}

for(j in 1: 46)

{

for(i in 1: N)

{

count[j,i]<-equals(county[i],j)

}

number[j]<-sum(count[j,])

PCHV[j]<-sum(PH[j,])/number[j]

}

}

#DATA latest

#INITIALS

list(taub=0.001, beta=c(0,0,0),Circum1=0,Urb1=0,STI1=0, Ever=0, edu1=c(NA,0,0,0),marital=c(NA,0,0,0,0),Fsex=c(NA,0,0,0,0),perceive=c(NA,0,0,0),b=c(0,0,0,0,0,0,0,0,0,0,0,0,0,0,0,0,0,0,0,0),omega.spatial1=0.01,U=c(0, 0, 0, 0, 0, 0, 0, 0, 0, 0, 0, 0, 0, 0, 0, 0, 0, 0, 0, 0, 0, 0, 0, 0, 0, 0, 0, 0, 0, 0, 0, 0, 0, 0, 0, 0, 0, 0, 0, 0, 0, 0, 0, 0, 0 ,0), tau.V=0.01,V=c(0, 0, 0, 0, 0, 0, 0, 0, 0, 0, 0, 0, 0, 0, 0, 0, 0, 0, 0, 0, 0, 0, 0, 0, 0, 0, 0, 0, 0, 0, 0, 0, 0, 0, 0, 0, 0, 0, 0, 0, 0, 0, 0, 0, 0 ,0))
